# A whole-food, plant-based randomized controlled trial in metastatic breast cancer: weight, cardiometabolic, and hormonal outcomes

**DOI:** 10.1007/s10549-024-07266-1

**Published:** 2024-03-06

**Authors:** Thomas M. Campbell, Erin K. Campbell, Eva Culakova, Lisa M. Blanchard, Nellie Wixom, Joseph J. Guido, James Fetten, Alissa Huston, Michelle Shayne, Michelle C. Janelsins, Karen M. Mustian, Richard G. Moore, Luke J. Peppone

**Affiliations:** 1 Department of Family Medicine, University of Rochester Medical Center, 777 South Clinton Ave, Rochester, NY 14620 USA; 2 Department of Public Health Sciences, University of Rochester Medical Center, Rochester, NY USA; 3 Department of Surgery, Cancer Control, University of Rochester Medical Center, Rochester, NY USA; 4 Clinical Research Center, University of Rochester Medical Center, Rochester, NY USA; 5 Memorial Sloan Kettering Cancer Center, Westchester, NY USA; 6 Department of Medicine, Hematology/Oncology, University of Rochester Medical Center, Rochester, NY USA; 7 Department of Obstetrics and Gynecology, University of Rochester Medical Center, Rochester, NY USA

**Keywords:** Diet, Nutrition, Breast cancer, Plant-based diet, Vegan diet, Obesity

## Abstract

**Purpose:**

Breast cancer treatment is associated with weight gain, and obesity and its related cardiometabolic and hormonal risk factors have been associated with poorer outcomes. Dietary intervention may address these risk factors, but limited research has been done in the setting of metastatic breast cancer requiring systemic therapy.

**Methods:**

Women with metastatic breast cancer on stable treatment were randomized 2:1 to an 8-week intervention (*n* = 21) or control (*n* = 11). The intervention included weekly assessment visits and an ad libitum whole-food, plant-based (WFPB) diet with provided meals. Cardiometabolic, hormonal, and cancer markers were assessed at baseline, 4 weeks, and 8 weeks.

**Results:**

Within the intervention group, mean weight decreased by 6.6% (*p* < 0.01) after 8 weeks. Fasting insulin decreased from 16.8 uIU/L to 11.2 uIU/L (*p* < 0.01), concurrent with significantly reduced insulin resistance. Total cholesterol decreased from 193.6 mg/dL to 159 mg/dL (*p* < 0.01), and low-density lipoprotein (LDL) cholesterol decreased from 104.6 mg/dL to 82.2 mg/dL (*p* < 0.01). Total testosterone was unchanged, but free testosterone trended lower within the intervention group (*p* = 0.08) as sex hormone binding globulin increased from 74.3 nmol/L to 98.2 nmol/L (*p* < 0.01). There were no significant differences in cancer progression markers at week 8, although mean CA 15-3, CA 27.29, and CEA were lower in the intervention group (*p* = 0.53, *p* = 0.23, and *p* = 0.54, respectively) compared to control, when adjusted for baseline.

**Conclusion:**

WFPB dietary changes during treatment for metastatic breast cancer are well tolerated and significantly improve weight, cardiometabolic and hormonal parameters. Longer studies are warranted to assess the durability of changes.

*Trial registration* First registered at Clinicaltrials.gov (NCT03045289) on February 7, 2017.

## Background

While many anti-neoplastic treatments are associated with weight loss, treatment for breast cancer (BC) is consistently associated with weight gain [1]. A 1997 review reported that significant weight gain occurs in 50% to 96% of women receiving chemotherapy for early stage BC, with a common weight gain of five to 13.6 pounds [2]. Little has changed since then [3]. Excess weight and weight gain remain common even through advanced BC. Among women receiving chemotherapy for metastatic disease, rates of obesity are comparable to or even higher than rates of obesity in the general population [4].

Obesity at diagnosis as well as excess weight gain after diagnosis has been associated with both BC-specific mortality and overall mortality [5–9]. In addition, obesity and its related cardiometabolic comorbidities contribute to higher symptom burden and reduced quality of life [10, 11]. Given this, it is not surprising that one survey found > 90% of patients with breast cancer who also have overweight or obesity reported being “somewhat” or “very” concerned about their weight [3].

Excess weight is often comorbid with elevated insulin and insulin resistance, blood glucose, cholesterol, sex hormones, and IGF-1. These may independently worsen risk of BC progression and mortality as well as reduce quality of life [12, 13]. Beyond cancer, it is well established that several of these comorbidities are risk factors for cardiovascular events [14], and cardiovascular disease is one of the leading causes of mortality (> 40%) among BC survivors [15].

**Fig. 1:**
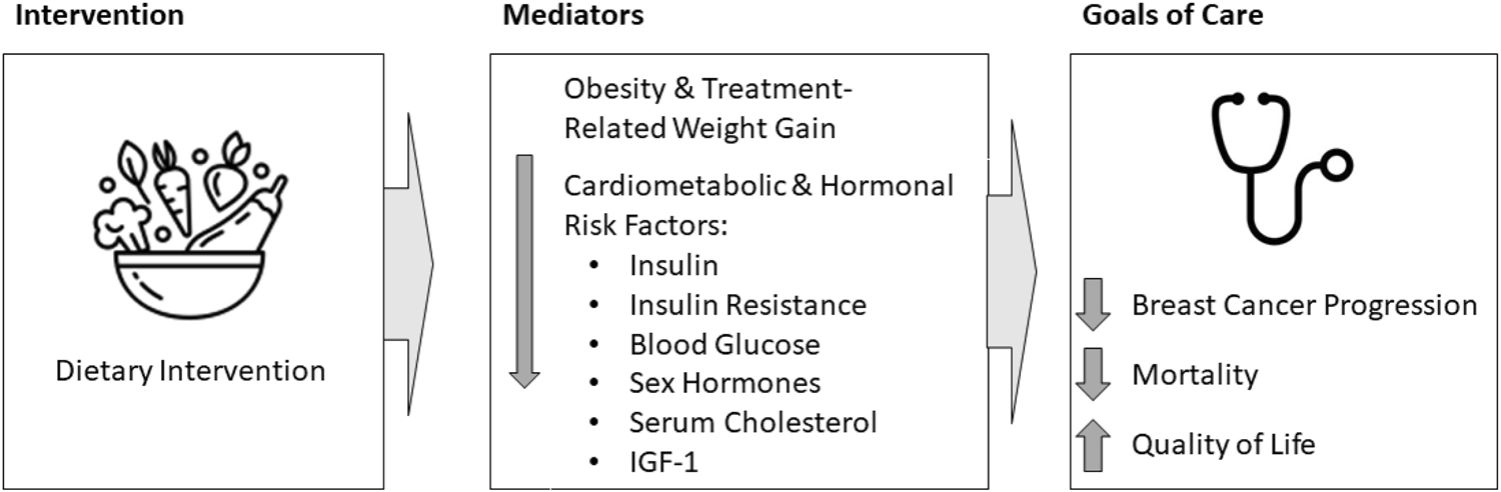
Potential mediators connecting dietary intake with goals of care. Dietary intervention may affect breast cancer-related goals of care through several potential mediators, some of which are shown here. This article details how a whole-food, plant-based intervention affects these mediators. Feasibility of the intervention and its effect on quality of life outcomes are published separately [42]

Dietary therapy can affect both obesity and its related cardiometabolic and hormonal risk factors (Fig. 1). A plant-predominant dietary pattern, lower in processed foods, is commonly recommended by many organizations, including the American Institute for Cancer Research [16] and the American Cancer Society [17]. At the end of the spectrum of plant-predominant dietary patterns is a whole-food, plant-based (WFPB) diet that minimizes or entirely avoids animal-based foods, highly processed foods, added fats and sugars. Interventions integrating this type of dietary approach have resulted in substantial weight loss [18, 19], regression of coronary atherosclerosis [20, 21], lowered cholesterol [22] and blood pressure [23] as well as reduced insulin resistance [24].

Patients with breast cancer, frequently concerned about their weight, are highly interested in nutrition information [25, 26]. Unfortunately, only limited research has investigated how dietary intervention affects BC-related outcomes [27, 28]. The findings of two large interventions [29, 30] suggest that weight loss, or diet and lifestyle change large enough to produce weight loss, may be necessary to impact cancer outcomes. Numerous other diet and lifestyle interventions have targeted weight loss among subjects with early stage breast cancer [31–40], but these studies have not been large enough or long enough to determine the effect of weight loss on recurrence or mortality and usually enrolled cancer survivors who already completed treatment.

Women with metastatic breast cancer on systemic therapy have largely been excluded from dietary intervention research, but with improved survival rates and an aging population, there is predicted to be 169,000 women living with metastatic breast cancer by 2025, up from 140,000 in 2018 [41]. Cancer burden is more easily tracked in metastatic breast cancer and there is a far higher risk of cancer progression and mortality compared to earlier stages. This presents an opportunity to understand how diet and lifestyle interventions may affect cancer-related outcomes within a shorter timeframe.

Given this background, we designed a pilot study to explore the feasibility and preliminary effects of a whole-food, plant-based dietary intervention in women with metastatic breast cancer. Findings relating to feasibility and effects on quality of life are published separately, while this report focuses on weight, cardiometabolic, and hormonal biomarkers.

## Methods

Women with metastatic breast cancer were recruited between February 2018 to March 2022 from oncology clinics at the University of Rochester Medical Center (URMC) and by flyers and announcements at local support groups in Rochester, NY. Women with stage 4 breast cancer with any ER/PR/HER2 status who were expected to live at least 6 months and who were on a stable treatment regimen for the past 6 weeks, with no planned treatment changes in the near future, were eligible for the study. Exclusions included inability to tolerate a normal diet, an active malabsorption syndrome or eating disorder, uncontrolled diarrhea, recent consumption of a vegan diet, major surgery within 2 months, current insulin, sulfonylurea, or warfarin use, glomerular filtration rate (GFR) < 30 mL/min/1.73 m^2^ or serum potassium > 5.3 mmol/L on two lab tests within 90 days, current smoking, illicit drug use, more than 7 alcoholic drinks per week, food intolerances to plant-based foods, or psychiatric disorder impairing ability to give consent.

Subjects were randomized 2:1 to two arms: whole food, plant-based (WFBP) intervention (*n* = 21) or usual diet control (*n* = 11). Subjects in the WFPB arm received 3 prepared meals and one side dish per day for 8 weeks, weekly assessment visits with the study physicians (TC and/or EKC), and a weekly phone call from a study physician (EC). Weekly assessment visits included education, coaching, and evaluation of adverse events or other medical changes. The ad libitum WFPB diet consisted of fruits, vegetables, whole grains, legumes, nuts and seeds. The diet excluded animal products and added oils/solid fats. Subjects were encouraged to eat as much and as often as they wanted to be comfortably full. They were encouraged to add their own food in addition to, or in place of, the provided food, as long as it was ‘on-plan.’ A daily multivitamin (Centrum Women) was provided to all subjects in both arms.

Subjects in the control arm continued their usual diets for 8 weeks and received phone calls from a study physician at weeks 2 and 6 to assess for adverse events and treatment changes. As an incentive to maintain participation, control subjects received condensed educational resources related to the WFPB diet and 2 weeks of prepared study meals after completing their final 8-week assessments.

### Testing procedures

All subjects had study visits and blood draws at baseline, week 4, and week 8. Weight and height were measured with subjects in light clothing, without shoes, on a Detecto Apex clinical digital scale with mechanical stadiometer. Blood pressure was measured with an automated blood pressure cuff with subjects seated quietly by themselves for 5 min before the monitor measured blood pressure three times, with 2 min between each measure. The average of the three blood pressures was recorded. Blood samples were drawn with subjects in a fasted state, in the morning, and tested using standard procedures at the CLIA certified URMC Clinical Laboratory. Blood tests included a complete metabolic panel, complete blood count, total and free testosterone, estradiol, sex hormone binding globulin (SHBG), dehydroepiandrosterone (DHEA), insulin and insulin-like growth factor-1 (IGF-1), insulin-like growth factor-1 binding protein (IGFBP-3), cholesterol panel, carcinoembryonic antigen (CEA), cancer antigen 27.29 (CA 27.29), and cancer antigen 15-3 (CA 15-3).

### Statistical analysis

Descriptive statistics (e.g., mean standard deviation [SD], *n*, percent) were used to evaluate distributions of patients’ clinical and sociodemographic variables to assess balance between treatment arm and control. For outcome measures (weight, BMI, cardiometabolic measures, biomarkers), the distributions were first evaluated graphically for normality and outliers. Mean, SD, and the range were calculated at baseline, 4 weeks, and 8 weeks by study arm to assess balance at baseline, within group changes at 4 and 8 weeks. Changes in outcome values from baseline to 4 and 8 weeks within each study arm were assessed by paired t test. Analysis of covariance model with arm as the main factor and corresponding baseline levels as the covariate was used to evaluate the effects of the WFPB intervention on the weight, BMI and cardiometabolic and biomarker outcomes at 8 weeks. The results were further evaluated in linear mixed effect model incorporating all three time points. Between-group difference in change from the baseline to 8 weeks was estimated by difference in marginal means at 8 weeks. The effect size (ES) was calculated as ratio of mean between group difference in change from baseline to the baseline SD. Additionally, since distribution of some of the markers did not fully follow Gaussian normal distribution, the within group and between group changes were also assessed by nonparametric tests. Results based on both parametric and nonparametric analyses were in agreement and supported the same conclusions. *p* values from the parametric analysis are shown. Statistical significance was set at two-sided alpha = 0.05 level. Data were analyzed using SAS/STAT software version 9.4 (SAS Inc, Cary, NC, USA).

## Results

**Fig. 2:**
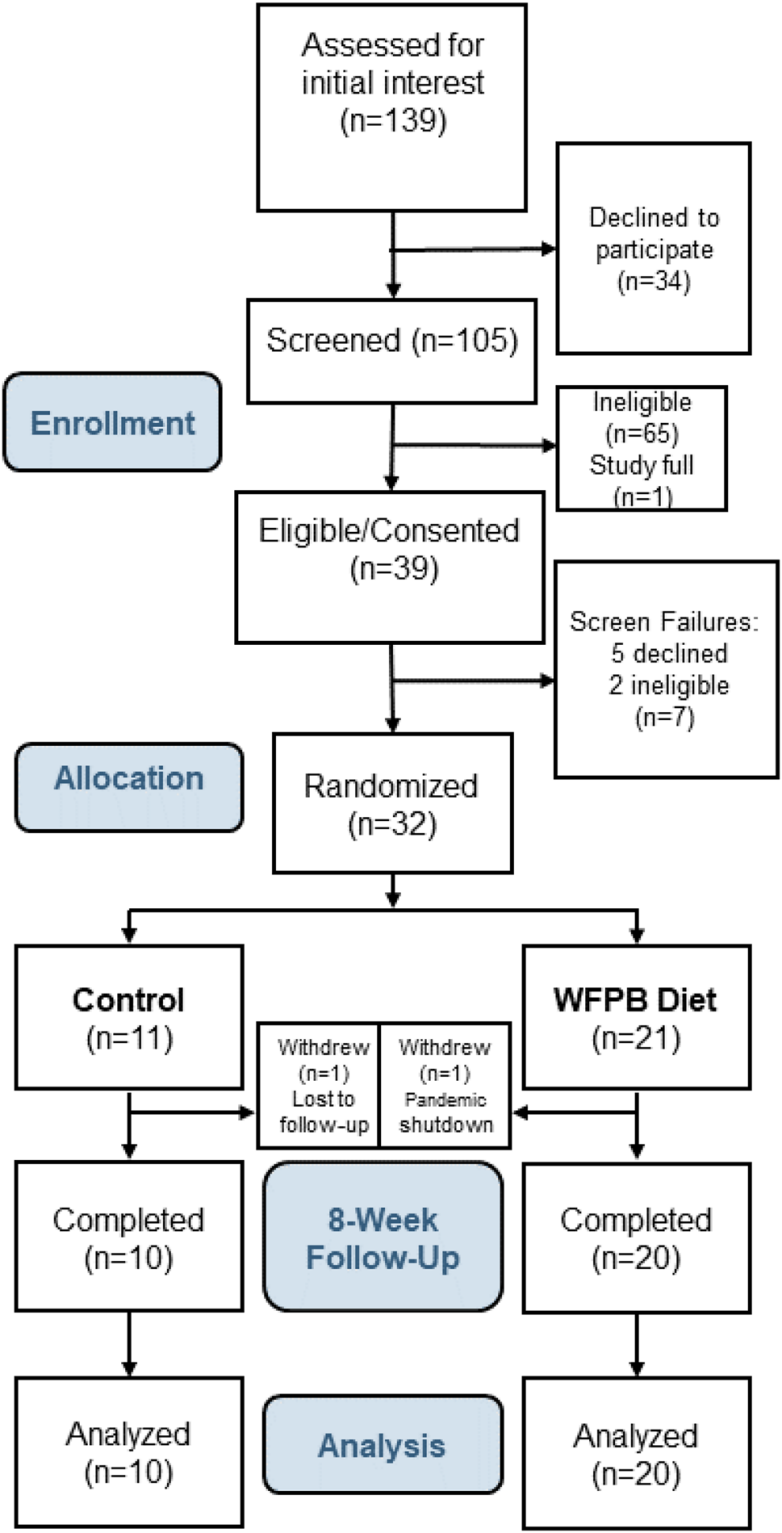
CONSORT diagram

Thirty of 32 (94%) randomized subjects completed their study participation. One subject was lost to follow-up immediately after being randomized to the control arm. One intervention subject was withdrawn by study investigators in March 2020 shortly after the baseline assessment due to the onset of the COVID-19 pandemic shutdown (Fig. 2). By our prespecified definition of compliance, 95% of subjects were compliant with the dietary prescription, and 100% of the subjects attended at least 6 of the 8 weekly assessment visits. Feasibility and dietary changes are detailed separately [42].

The characteristics of the 31 subjects who completed baseline assessments are shown in Table 1. Of these subjects, 29% had BMIs categorized as normal (BMI 18.5–24.9 kg/m^2^), 32.3% as overweight (BMI 25–29.9 kg/m^2^), and 38.7% as obese (BMI ≥ 30 kg/m^2^). Almost all had hormone receptor positive breast cancer, and the most common treatment regimen was a cyclin-dependent kinase 4/6 inhibitor and an aromatase inhibitor. The most common site of metastasis was bone.

**Table 1:** Baseline characteristics

		Control (10)	Intervention (21)
Age	Mean (SD)	64.2 (8.9)	59.1 (11)
Race	White, % (*n*)	100.0 (10)	90.5 (19)
	Black, % (*n*)	0	4.8 (1)
	No answer, % (*n*)	0	4.8 (1)
Ethnicity	Not Hispanic/Latino, % (*n*)	100.0 (10)	95.2 (20)
	No answer, % (*n*)	0	4.8 (1)
Marital Status	Married, % (*n*)	70.0 (7)	66.7 (14)
	Divorced, % (*n*)	20.0 (2)	14.3 (3)
	Single, % (*n*)	10.0 (1)	14.3 (3)
	Widowed, % (*n*)	0	4.8 (1)
Employment Status	Currently employed outside home, % (*n*)	30.0 (3)	28.6 (6)
	Self-employed, % (*n*)	0	9.5 (2)
	Retired, % (*n*)	40.0 (4)	19.0 (4)
	Disability, % (*n*)	10.0 (1)	14.3 (3)
	Homemaker, % (*n*)	20.0 (2)	19.0 (4)
	Not Working—Other, % (*n*)	0	9.5 (2)
BMI at Study Baseline	Mean, Kilograms/m^2^ (SD)	28.4 (4.4)	30.2 (7.2)
Age at First Breast Cancer Diagnosis	Mean (SD)	52.9 (11.7)	49.4 (10.9)
Years Elapsed Since First Diagnosis	Mean (SD)	11.2 (7.9)	9.7 (6.4)
Years Elapsed Since Diagnosis of Metastatic Breast Cancer	Mean (SD)	5.3 (6.0)	2.2 (1.8)
Hormone Receptor Status	ER + , % (*n*)	100.0 (10)	95.2 (20)
	PR + , % (*n*)	90.0 (9)	81.0 (17)
	HER2 + , % (*n*)	30.0 (3)	28.6 (6)
Location of Metastases	Bone, % (*n*)	70.0 (7)	90.5 (19)
	Lung, % (*n*)	40.0 (4)	38.1 (8)
	Brain, % (*n*)	10.0 (1)	14.3 (3)
	Liver, % (*n*)	20.0 (2)	4.8 (1)
	Other, % (*n*)	60.0 (6)	33.3 (7)
Cancer Therapy	Palbociclib, % (*n*)	30.0 (3)	47.6 (10)
	Abemaciclib, % (*n*)	10.0 (1)	9.5 (2)
	Ribociclib, % (*n*)	0	4.8 (1)
	Trastuzumab, % (*n*)	20.0 (2)	23.8 (5)
	Pertuzumab, % (*n*)	10.0 (1)	19.0 (4)
	Capecitabine, % (*n*)	10.0 (1)	4.8 (1)
	Letrozole, % (*n*)	30.0 (3)	61.9 (13)
	Anastrozole, % (*n*)	30.0 (3)	4.8 (1)
	Exemestane, % (*n*)	10.0 (1)	9.5 (2)
	Fulvestrant, % (*n*)	20.0 (2)	14.3 (3)
	Denosumab, % (*n*)	10.0 (1)	47.6 (10)
	Zoledronic acid, % (*n*)	0	4.8 (1)
	Leuprolide, % (*n*)	0	9.5 (2)

Results for 20 intervention and 10 control subjects with complete data are shown in Table 2. Mean weight among intervention subjects decreased from 177.5 lbs to 165.7 lbs at 8 weeks, or a 6.6% decrease, which represents an average loss of approximately 1.5 lbs a week. BMI decreased from 29.7 to 27.8 kg/m^2^. When adjusted for baseline, intervention subjects lost 9 lbs more than control subjects (*p* ≤ 0.01, effect size − 0.21) and lost 1.7 kg/m^2^ more from their BMI (*p* ≤ 0.01, effect size − 0.26). Concurrently, mean total cholesterol level decreased 17.7% and mean LDL cholesterol levels decreased 21.4% to 82.2 mg/dL within the intervention group. Compared to control, mean total cholesterol levels decreased by 35.3 mg/dL (*p* ≤ 0.01, effect size − 0.93) and mean LDL levels decreased by 23.5 mg/dL (*p* ≤ 0.01, effect size − 0.75) in the intervention group.

**Table 2:** Outcomes between baseline and 8 weeks in intervention and control groups

Outcome	Intervention diet		Usual diet control		Between group differences in change at week 8 (adjusted for baseline value)^d^		
	Baseline	Week 8	Baseline	Week 8	Diff	Effect size	*p* value
Weight (lbs)	177.5	165.7*	159.9	158.8	− 9.0	− 0.21	< 0.01
BMI	29.7	27.8*	28.4	28.2	− 1.7	− 0.25	< 0.01
*Cardiometabolic outcomes*							
Total Cholesterol (mg/dL)^a^	193.6	159.4*	174.6	181.4	− 35.3	− 0.93	< 0.01
LDL Cholesterol (mg/dL)^a^	104.6	82.2*	92.3	97.9	− 23.5	− 0.75	< 0.01
Triglycerides (mg/dL)^a^	111.1	113.0	106.7	118.4	− 8.8	− 0.21	0.55
HDL (mg/dL)^a^	66.7	54.7*	60.8	59.8	− 9.3	− 0.60	< 0.01
Systolic BP (mmHg)^b^	113.2	110.3	111.0	113.3	− 3.9	− 0.33	0.28
Diastolic BP (mmHg)^b^	71.3	68.8	65.3	66.7	− 1.9	− 0.16	0.50
Glucose (mg/dL)	101.5	93.8	114.4	117.3	− 11.9	− 0.45	0.16
Insulin (uIU/L)	16.8	11.2*	11.4	12.1	− 3.8	− 0.39	0.12
HOMA-IR	4.4	2.7*	3.2	3.5	− 1.3	− 0.43	0.10
*Hormonal markers*							
Sex Hormone Binding Globulin (nmol/L)	74.3	98.2*	89.0	78.9*	33.4	0.84	< 0.01
Total Testosterone (ng/dL)	23.7	23.7	16.8	15.6	2.7	0.19	0.21
Free Testosterone (ng/dL)	0.49	0.32	0.25	0.28	− 0.01	− 0.03	0.90
DHEA (ug/dL)	110.9	106.8	56.5	53.7	2.8	0.06	0.72
IGF-1 (ng/mL)	173.8	156.4*	150.5	144.7	− 7.9	− 0.16	0.38
IGFBP-3 (ng/mL)	4966	4874	4415	4418	− 23.7	− 0.02	0.92
*Blood counts*							
White Blood Cells (1000/uL)	3.7	3.3	4.8	4.7	− 0.7	− 0.37	0.06
Neutrophils (1000/uL)	2.0	1.9	3.0	2.8	− 0.4	− 0.25	0.16
Lymphocytes (1000/uL)	1.1	1.0	1.3	1.3	− 0.1	− 0.17	0.38
Hemoglobin (g/dL)	12.3	12.2	12.1	12.4	− 0.3	− 0.27	0.37
Platelet (1000/uL)	215	206	222	223	− 12.0	− 0.18	0.34
*Cancer progression markers*							
CA 27.29 (U/mL)^c^	25.7	24.6	84.9	97.5	− 5.3	− 0.08	0.23
CA 15-3 (U/mL)^c^	22.3	22.7	90.8	111.2	− 5.2	− 0.07	0.53
CEA (ng/mL)	3.1	3.2	7.9	10.2	− 0.5	− 0.07	0.54

Values are means

^*^p < 0.05 for within-group change

^a^One intervention subject was excluded from the cholesterol analysis due to having stopped her cholesterol medication midway through the study

^b^One intervention subject was excluded from the blood pressure analysis due to missing a baseline measurement

^c^One intervention subject was excluded from the cancer marker analysis due to being an extreme outlier

^d^The mean between-group difference in change from the baseline to 8 weeks was estimated by marginal means calculated via linear mixed effect model

Blood pressure was at optimal levels in both groups at baseline with no statistically significant changes during the intervention, although blood pressure trended lower in the intervention group. Compared to baseline, mean fasting blood glucose levels were lower within the intervention group at 8 weeks, but this did not meet significance (*p* = 0.11). Although baseline insulin was within the normal range (3–25 uIU/mL), decreases were noted within the intervention group, from 16.8 uIU/mL to 11.2 uIU/mL (*p* < 0.01). Insulin resistance, as calculated by HOMA-IR, decreased in the intervention group, from 4.4 to 2.7 (*p* = 0.01).

Sex hormone binding globulin increased within the intervention group (*p* ≤ 0.01), likely related to weight loss in this group [43]. SHBG happened to decrease within the control group without a known cause (*p* = 0.05). When adjusted for baseline, the intervention group saw a 33.4 nmol/L increase in sex hormone binding globulin compared to control (*p* ≤ 0.01, effect size 0.84). DHEA was not statistically different in either group at 8 weeks. Accordingly, while changes in total testosterone did not reach statistical significance in either group, free testosterone was lower within the intervention group at 8 weeks, though this was not statistically significant (*p* = 0.08). Estradiol was undetectable at baseline in the majority of subjects given that natural or chemically induced menopause was common, as reflected by the fact that 74% of subjects were on an aromatase inhibitor. Insulin-like growth factor 1 (IGF-1) significantly decreased by 10% within the intervention group (*p* = 0.01), but the between-group difference was not statistically significant.

White blood cells (WBCs) were slightly lower at 8 weeks within the intervention group, a difference that approached statistical significance (*p* = 0.06) when compared to the control group. Most participants were on therapy that strongly affects white blood cell counts, and blood draws were not done at the same point in each treatment cycle. The change in WBCs did not appear to be clinically significant, and a mechanistic relationship to the dietary intervention was not established. Hemoglobin and platelets were not significantly different within either group or between the groups from baseline to 8 weeks. There was no statistically significant difference in changes between the groups in serum sodium, potassium chloride, bicarbonate, and calcium, or alanine transaminase (ALT), aspartate aminotransferase (AST), or serum total protein. Kidney function as measured by creatinine and the estimated glomerular filtration rate were not significantly different, but urea nitrogen was significantly lower at 8 weeks within the intervention group (*p* ≤ 0.01).

In the intervention group, the cancer markers CA 27.29, CA 15-3, and CEA were in the normal range at baseline and were not statistically significantly different at 8 weeks within the intervention group or between the two groups.

Adverse events related to the intervention were infrequent and mild. Three intervention subjects had grade 2 hypotension during the study with mild symptoms (Two on antihypertensives and one on an extensive pain control regimen) and were referred to their routine care providers for medication adjustments. One control subject experienced lightheadedness following a blood draw. Other adverse events (mild, transient neutropenia, aphthous ulcer, transient, mild hyponatremia) were deemed related to medications. One subject in each group had the dose of their primary cancer therapy reduced due to adverse events typical of their medication.

## Discussion

This is one of the first studies to demonstrate both feasibility and clinically important improvements from a dietary change in metastatic breast cancer patients receiving systemic therapy. Results from this study showed that our whole-food, plant-based dietary intervention promotes significant weight loss and improves several cardiometabolic and hormonal risk factors among women with metastatic breast cancer. Specifically, subjects in the intervention group, who had a baseline BMI of 29.7 kg/m^2^, lost 1–2 pounds a week and saw significant improvements, both clinically and statistically, in cholesterol, insulin, insulin resistance, sex hormone binding globulin, and IGF-1.

Several other nutrition and lifestyle trials for cancer survivors, conducted after subjects had completed their primary cancer treatment, have demonstrated feasibility and weight loss, but most show lower or significantly slower weight loss than this trial [31–40]. Weight loss in this study reflects the large nutritional changes achieved, described in a separate report [42]. While this amount of weight loss may be larger and/or faster compared to previous cancer interventions, the rate of weight loss is consistent with other interventions using whole-food, plant-based diets, even when no prepared food is provided [18, 44]. It is also consistent with recommendations for individuals with excess weight in the general population to target 1 to 2 pounds of weight loss per week during their weight loss efforts [45].

The weight loss was due to large, intentional dietary changes, without signs or symptoms of concurrently progressing disease or cachexia, and it was achieved without portion or calorie restriction or mandated exercise. Coaching included frequent recommendations to eat greater volumes of food and eat more frequently, while choosing foods that were ‘on plan.’ When comparing the final 3-day food diary with the baseline 3-day food diary, intervention subjects had 16% greater dietary intake (solid plus liquid intake) in terms of weight but consumed 26% fewer calories, suggesting significantly reduced calorie density of the study diet compared to their baseline diets.

Consistent with weight loss, the cardiometabolic and hormonal milieu improved within the intervention group. These changes likely relate to a convergence of mechanisms. Weight loss has been shown to increase SHBG [43, 46], lower free androgens [43], and improve cardiometabolic risk factors including insulin, insulin resistance, and cholesterol levels [47, 48]. However, the specific plant-based dietary composition also likely played a role. Dietary patterns with substantial increases in dietary fiber and substantial reductions in saturated fat and/or animal protein have been tested in various human trials and found to modulate serum cholesterol [49, 50], insulin resistance [51, 52], sex hormones [53–55], and IGF-1 [56].

Whether this weight loss or risk factor modification improves cancer-specific progression or mortality is unknown. Barnard et al. [57] found that, in post-menopausal women with overweight or obesity, a 2-week intervention consisting of an ad libitum whole-food, plant-predominant diet and exercise resulted in significantly reduced estradiol, insulin, and IGF-1. When comparing subjects’ pre- and post-intervention serum in vitro, using 3 estrogen receptor-positive breast cancer cell lines, there was a significant decrease in cell growth and increase in apoptosis concurrent with improvements in biomarkers following the diet and lifestyle intervention.

In our study, there were no significant changes in cancer markers at 8 weeks, although the intervention group showed a more stable trend compared to the control group. Among participants who did not have elevated cancer markers during the study, 50% of control participants and 46% of intervention participants previously had elevated markers, suggesting a relatively similar percentage of participants in both groups who had cancer markers that reflected cancer activity. The normal baseline levels among the intervention group, along with our small sample size, limited our ability to detect larger changes. The differences in baseline levels between the two groups were made more likely by the possibility of wide variation in cancer marker levels combined with our smaller sample size. In addition to these limitations, the short duration of the trial makes changes in cancer markers more difficult to interpret given the possibility of spurious results in the first 4–6 weeks following therapy changes [58].

This randomized controlled trial has numerous limitations and strengths. The study duration limits our ability to know whether these findings are sustainable, and whether these findings affect risks of cancer progression or mortality. This brief intervention was not intended to support long-term behavior change, and participants’ outcomes will not be followed over time. The size of the study, particularly the smaller control group, limits our ability to detect smaller differences in outcomes. In addition, the lack of racial diversity as well as the overrepresentation of hormone-receptor positive breast cancer both limit generalizability. The control group was not matched in terms of time with, and attention from, study staff, making it harder to isolate the effects of the dietary changes from the effects of the overall intervention. Strengths of the study include the very large dietary changes achieved and high retention rate, presumably related to the intensity of the intervention and the provided food.

## Conclusion

Our whole-food, plant-based intervention among women with metastatic breast cancer is feasible and results in clinically significant improvements in weight and related cardiometabolic and hormonal risk factors. This is one of the first RCTs to demonstrate that dietary changes during systemic treatment are well tolerated and result in these clinically important improvements. This is particularly relevant for this population, which is highly interested in nutrition and concerned with treatment-related weight gain, its comorbid conditions, and its implications for cancer-related outcomes. Trials of longer duration are required to understand the sustainability of these findings as well as their effects on cancer progression and mortality.

## Data Availability

The data underlying this article are available by request at https://gitlab-public.circ.rochester.edu/WFPB-breast-cancer/biomarkers.

## Abbreviations

BC: Breast cancer
CA 27.29: Cancer antigen 27.29
CA 15-3: Cancer antigen 15-3
CEA: Carcinoembryonic antigen
DHEA: Dehydroepiandrosterone
HOMA-IR: Homeostatic Model Assessment of Insulin Resistance
IGF-1: Insulin-like growth factor-1
IGFBP-3: Insulin-like growth factor-1 binding protein-3
LDL: Low-density lipoprotein
SHBG: Sex hormone binding globulin
WFPB: Whole-food, plant-based
